# Role of LncRNA MSTRG.20890.1 in Hair Follicle Development of Cashmere Goats

**DOI:** 10.3390/genes15111392

**Published:** 2024-10-29

**Authors:** Min Wang, Rong Ma, Qing Ma, Bingjie Ma, Fangzheng Shang, Qi Lv, Zhiying Wang, Ruijun Wang, Rui Su, Yanhong Zhao, Yanjun Zhang

**Affiliations:** 1 College of Animal Science, Inner Mongolia Agricultural University, Hohhot 010018, China; wm20240906@163.com (M.W.); marong202406@163.com (R.M.);; 2 Key Laboratory of Mutton Sheep Genetics and Breeding, Ministry of Agriculture, Hohhot 010018, China; 3 Laboratory of Goat and Sheep Genetics, Breeding and Reproduction in Inner Mongolia Autonomous Region, Hohhot 010018, China

**Keywords:** cashmere goat, hair follicle, lncRNA, morphogenesis, dermal fibroblast

## Abstract

Background: The cashmere goat is a biological resource that mainly produces cashmere. Cashmere has a soft hand feel and good luster, with high economic value. The quality and yield of cashmere are determined by the process of hair follicle development during the embryonic period. Methods: In this study, the skin of the Inner Mongolia cashmere goat at different embryonic stages (45, 55, 65, and 75d) was collected, and the differentially expressed lncRNA MSTRG.20890.1 at 75d was obtained by screening. Dual luciferase reporter gene system, qRT-PCR, and EDU experiments were used to verify further the regulatory role and molecular mechanism of the lncRNA in dermal fibroblasts. Results: Based on the transcriptome database of Inner Mongolia cashmere goat skin at different embryonic stages, which was previously constructed by our group, according to the characteristics of hair follicle development in the embryonic stage, we screened out the lncRNA MSTRG.20890.1 that was down-expressed on the 75-SHFINI day of the embryonic stage. We found that lncRNA MSTRG.20890.1 was mainly located in the cytoplasm of cells, and it could inhibit the proliferation and directional migration of dermal fibroblasts through the chi-miR-24-3p/ADAMTS3 signaling axis, thereby inhibiting the formation of dermal papilla structure at embryonic stage. Conclusions: This study revealed that lncRNA MSTRG.20890.1 regulated secondary hair follicle morphogenesis and development in cashmere goats through the chi-miR-24-3p/ADAMTS3 signaling axis.

## 1. Introduction

Cashmere goats originated from wild goats. After long-term domestication by human beings, they gradually developed into a kind of goat that adapted to local living conditions and mainly produced cashmere. Cashmere goats mainly survive in arid, semi-arid, desert, and semi-desert areas between 25° N and 55° N latitude and 40° E and 125° E longitude, as well as in high and cold areas such as plateaus, hills, and mountainous areas at an altitude of 1500 m to 4500 m. They are mainly distributed in Asian countries such as China, Russia, ongolia, and Iran. There are multiple cashmere goat breeds worldwide, such as Orenburg goats, Inner Mongolia cashmere goats and Liaoning cashmere goats, etc. Among them, the cashmere produced by Inner Mongolia cashmere goats (Hohhot, China) has a soft hand feel and good luster [1]. It is favored in the international market. Its cashmere products are known for their high quality and good comfort and have strong competitiveness in the international market.

China is the world’s largest cashmere goat feeding country and cashmere production country. According to statistics, in 2018, China’s cashmere output was about 15,437.76 t, accounting for more than 2/3 of the world’s total; the export of cashmere was 3212 t, accounting for more than 20% of the national total output. Inner Mongolia Autonomous Region is a superior production area of cashmere goats in China. The cashmere it produces has a soft feel and good quality. By the end of 2018, the production of cashmere in the Inner Mongolia Autonomous Region reached 6606.83 t. The cashmere produced in this area accounts for 42.8% of the total production of cashmere in China and one-third of the total production of cashmere in the world. The cashmere goat industry provides employment opportunities and an important source of income for many farmers and herders, especially in areas such as the Inner Mongolia Autonomous Region where husbandry is dominant. The development of the cashmere goat breeding industry plays an irreplaceable role in ensuring the lives of residents and promoting the development of the agricultural economy. Increasing the yield and quality of cashmere is the main channel for farmers and herdsmen to increase income and the rapid development of cashmere processing enterprises, which can effectively promote the development of the local economy and society. The hair follicles of cashmere goats are mainly classified as primary hair follicles and secondary hair follicles. Among them, cashmere grown from secondary hair follicles is one of the important textile raw materials in the textile industry [2,3]. The process of hair follicle development commences in the embryonic stage and is accomplished after birth [4]. At 45 days of the embryonic stage of cashmere goats, the fetal skin forms a complete epidermal structure, and the hair follicle formation is not yet initiated (45-EPI). Around day 55 of the embryonic stage, primary hair follicles begin to form, and the keratinocytes in the basal layer of the epidermis are arranged together in a palisade pattern to form the hair bud structure of the primary hair follicles (55-PHFBUD). By approximately day 65, the hair bud structure of the primary hair follicle grows downward into the dermis of the skin (65-PHFDERM). At around 75 embryonic days, secondary hair follicle morphology begins to occur (75-SHFINI) [2,5].

Long non-coding RNA (lncRNA) is a type of non-coding RNA (ncRNA) that has a transcription length of more than 200 nucleotides and lacks protein-coding ability [6,7]. Unlike mRNAs, lncRNAs have unique modes of transcription, processing, and modification [8]. Recent research has indicated that although lncRNA does not possess the function of encoding proteins, it indirectly regulates the expression of protein-coding genes in aspects of epigenetic regulation, transcriptional regulation, and post-transcriptional regulation [9,10,11]. Among them, the regulatory mechanism of lncRNA can be preliminarily predicted based on its location in cells. In the cytoplasm, lncRNAs can often play a regulatory role as a competing endogenous RNA (ceRNA). Zhao [12] analyzed different growth stages of Aohan fine wool sheep skin and finally found 461 differentially expressed lncRNAs. Among them, lncRNA MSTRG.223165 can participate in the hair follicle development process as a ceRNA of miR-21. During hair follicle development, lncRNA-XIST promotes *Shh* expression by binding to miR-424, thus promoting hair follicle regeneration and development [13]. lncRNA-H19 promotes the proliferation and vitality of dermal papilla cells through the chi-miR-214-3p/β-catenin signaling axis [14]. The above results indicate that lncRNA actively participates in hair follicle development. Nevertheless, the role that new lncRNAs play in the regulation of hair follicle development remains largely unknown.

To further explore the regulatory role of lncRNA in hair follicle development, according to the characteristics of hair follicle morphogenesis and development in cashmere goats, lncRNA MSTRG.20890.1, which is related to secondary hair follicle morphogenesis and differentially expressed at 75-SHFINI days of embryonic stage, was screened out. By bioinformatics analysis, it was found that this lncRNA was mainly expressed in the cytoplasm and may play a role through the chi-miR-24-3p/ADAMTS3 signaling axis. Subsequently, the signaling axis was verified in dermal fibroblasts. Finally, our research results indicate that lncRNA MSTRG.20890.1 regulates the proliferation and migration of dermal fibroblasts by competitively binding to *ADAMTS3* with chi-miR-24-3p, thereby inhibiting the formation of the dermal papilla structure. At the same time, we found that this inhibitory effect on the proliferation and migration of dermal fibroblasts may be achieved by inhibiting cell proliferation-related genes and reducing the proportion of cells in the S phase. In conclusion, we believe that lncRNA MSTRG.20890.1 regulates the proliferation and migration of dermal fibroblasts through the chi-miR-24-3p/ADAMTS3 signal axis, thereby inhibiting the formation of the dermal papilla structure and the morphogenesis of secondary hair follicles.

## 2. Materials and Methods

### 2.1. Ethics Approval and Consent to Participate

The cashmere goat farm adheres to the relevant stipulations of the Chinese national standard “Laboratory Animal Environment and Facilities” [15]. All samples were collected following the “International Guidelines for Biomedical Research Involving Animals”, and were sanctioned by the Special Committee for Research and Academic Ethics of Inner Mongolia Agricultural University (Approval No. [2020] 056).

### 2.2. Sample Collection

The experimental animals in this study were Inner Mongolia cashmere goats (Albas type), which mainly live in the western region of Inner Mongolia Autonomous Region in China (106°41′–108°54′ E, 38°18′–41°11′ N). The average altitude of this area is 1304 m, the average annual sunshine hours are 3000 h, and it belongs to the temperate continental climate. The winter in this region is long and cold while the summer is warm but short. Twelve 3-year-old pregnant Inner Mongolia cashmere goat ewes with the same feeding conditions were selected. Skin samples of about 1 cm^2^ were collected from 45, 55, 65, and 75 d fetuses based on the mating records. Three fetuses were collected in each period. Subsequently, the collected samples were stored in liquid nitrogen.

### 2.3. Screening and Identification of Key LncRNAs in Secondary Hair Follicle Morphogenesis

In the previous research, our team p—erformed transcriptome sequencing on 12 skin samples of Inner Mongolia cashmere goats at four different embryonic periods (45 days, 55 days, 65 days, and 75 days), and obtained lncRNA expression profiles in skin samples at four different embryonic stages [16]. First, we detected the quality of the total RNA samples extracted by the Trizol reagent, and the detections of 12 samples met the sequencing standards. Subsequently, paired-end sequencing was performed using Illumina Hiseq 4000 to obtain the raw date. We used Bowtie [17] and Hisat2 [18] tools to compare the data with the reference genome, the reads were assembled and transcripts utilizing Stringtie, and we used CPC [19] and CNCI [20] to predict the coding ability of the RNA. Finally, RNAs with transcripts longer than 200 bp and a CPC score ≤ 0.5 and a CNCI score ≤ 0 were considered lncRNAs for subsequent studies. (CPC score = Sum of coding feature weights − Sum of non-coding feature weights; CNCI score = the FPKM value of the target gene/the average FPKM value of all coding genes in the reference sample).

We Screened for differentially expressed lncRNA associated with secondary hair follicle morphogenesis based on embryonic hair follicle development characteristics. The three comparison groups of 55-PHFBUD vs. 45-EPI, 65-PHFDERM vs. 45-EPI, and 65-PHFDERM vs. 55-PHFBUD were used as Stage A related to the development of primary hair follicles, and the three comparison groups of 75-SHFINI vs. 45-EPI, 75-SHFINI vs. 55-PHFBUD, and 75-SHFINI vs. 65-PHFDERM were used as the Stage B related to the development of both primary and secondary hair follicles. The common part of Stage B and Stage A was removed from Stage B, and after removing the intersection part from Stage B, the remaining part was used as the important lncRNAs related to the morphogenesis and development of secondary hair follicles. The R language was used to visualize the screened data. The screening criterion of differentially expressed lncRNAs was based on the FPKM value [21], the edge software was used for differential expression analysis, and the conditions of screening were |log2(Foldchange)| ≤ 1 and *p*-value ≤ 0.05. (FPKM = Total exon fragments/(Mapped reads × Exon length)).

### 2.4. Construction of Interference/Overexpression Cell Lines

lncRNA MSTRG.20890.1 interference plasmid, *ADAMTS3* interference plasmid, and chi-miR-24-3p interference/overexpression plasmid were prepared by Hanheng Biological Technology Co., Ltd. (Shanghai, China). The lentiviral transfection was performed according to the manufacturer’s instructions. Cells were cultured in basal medium containing the optimal concentration of polybrene for 4 h, and then the culture medium was replaced and lentivirus was added for transfection. After transfection, Puromycin was added for resistance screening.

### 2.5. Isolation of Cytoplasmic and Nuclear RNA

LncLocator 1.0 (http://www.csbio.sjtu.edu.cn/bioinf/lncLocator/; access on 9 July 2024) was used to predict the location of lncRNA MSTRG.20890.1 in cells [22]. At the same time, the Cytoplasmic & Nuclear RNA Purification Kit (Norgen Biotek, Thorold, ON, Canada) was used to extract and purify the cytoplasmic RNA and nuclear RNA of dermal fibroblasts.

### 2.6. Dual-Luciferase Report Detection

Targetscan [23] and miRanda [24] were used to predict targeting relationship. Subsequently, we chose the chi-miR-24-3p/ADAMTS3 signaling axis as the candidate target of lncRNA MSTRG.20890.1. The dual-luciferase reporter gene system was used to detect the targeting relationship between miRNA and other RNAs. In 293T cells, the chi-miR-24-3p mimic was co-transfected with psiCHECK2-MSTRG.20890.1-WT/MUT and psiCHECK2-ADAMTS3-WT/MUT respectively using the LipoFiter transfection reagent (Hanheng, Shanghai, China). After transfection for 48 h, luciferase activity was measured using a dual luciferase reporter system (Promega, Madison, WI, USA).

### 2.7. Cell Proliferation Assays

Cell proliferation was detected by CCK8 and EDU methods. CCK8 method: The capacity for cellular proliferation was measured using the Cell Counting Kit-8 (CCK-8) (Solarbio, Beijing, China) according to the manufacturer’s instructions. The optical density was determined with a microplate reader at a wavelength of 450 nm. EdU assay: The EdU assay was performed based on the instructions of the BeyoClick™ EdU-555 Cell Proliferation Detection Kit (Beyotime, Shanghai, China). The proliferation rate of dermal fibroblasts in each group was calculated as the ratio of EdU-labeled proliferating cells to all Hoechst-labeled cells.

### 2.8. Cell Apoptosis Detection

According to the manufacturer’s instructions, the apoptosis of cells was determined using the Annexin V-APC/PI Apoptosis Detection Kit (Elabscience Biotechnology, Wuhan, China). Single-cell suspension was prepared, and 100 μL of 1× Annexin V Binding Buffer, 2.5 μL of Annexin V-APC Reagent, and 2.5 μL of PI Reagent were added successively; the cells were resuspended and incubated in the dark for 20 min. Finally, flow cytometry was used to detect cell apoptosis.

### 2.9. DNA Staining

According to the manufacturer’s instructions, the cell cycle was determined by the DNA content quantification method (Solarbio, Beijing, China). Cells were gathered and supplemented with 70% pre-cooled ethanol. The cells were resuspended and placed in a refrigerator at 4 °C for fixation. After 24 h, the RNase A solution was added to the cell sediment and the cells were resuspended and incubated for 30 min at 37 °C in a water bath. Subsequently, 400 μL of PI staining solution was added and incubated at 4 °C in the dark for 30 min.

### 2.10. Scrape Motility Assay

The scratch motility assay was performed by scraping the cell monolayer with a sterile 200 µL pipette tip and taking pictures of the scratched area using a microscope. Cell scratch images were collected at 0 h and 24 h after scratch. Image-pro Plus v6.0 image analysis software was used to analyze the images.

### 2.11. Statistical Analyses

The statistical analysis mainly used SPSS 26.0 (SPSS, Chicago, IL, USA) and GraphPad Prism 8.0 (GraphPad Software Inc., La Jolla, CA, USA). All data are presented as the mean ± standard deviation from at least three independent repeated experiments. All data were tested for normal distribution. Student’s *t*-tests and Wilcoxon signed rank test were used for analysis. A *p*-value < 0.05 from a two-tailed test was considered significant.

## 3. Results

### 3.1. Important LncRNA Screening for Secondary Hair Follicle Morphogenesis

**Figure 1:**
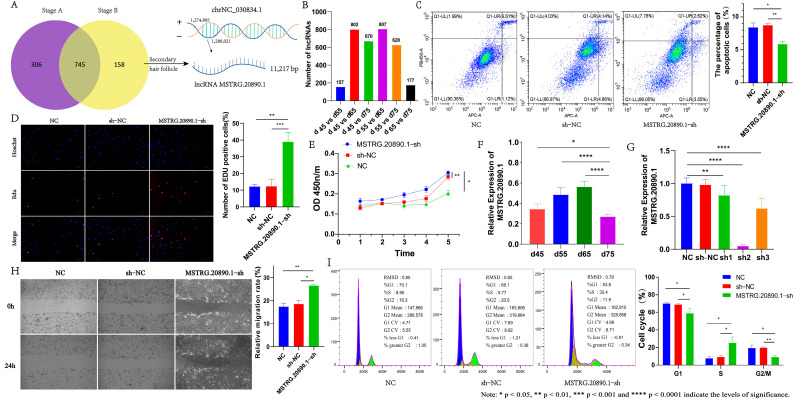
Functional analysis of lncRNA MSTRG.20890.1 in dermal fibroblasts. (**A**) Screening of lncRNA MSTRG.20890.1 related to secondary hair follicle morphogenesis. (**B**) Expression of lncRNA in different treatment groups. (**C**) The apoptosis of dermal fibroblasts was detected after lncRNA MSTRG.20890.1 interference. (**D**) EDU was used to detect the proliferation of lncRNA MSTRG.20890.1-sh cell line. (**E**) CCK8 was used to detect the proliferation of lncRNA MSTRG.20890.1-sh cell line. (**F**) Expression of lncRNA MSTRG.20890.1 in skin samples at different embryonic periods. (**G**) Screening of lncRNA MSTRG.20890.1 interference vector. (**H**) Migration ability of lncRNA MSTRG.20890.1-sh cell line. (**I**) Cell cycle determination of lncRNA MSTRG.20890.1-sh cell line.

Based on the transcriptome database of Inner Mongolia cashmere goat skin at different embryonic stages (45, 55, 65, and 75 days), which our group had constructed in the previous period [16], we identified a total of 1209 differentially expressed lncRNAs, among which there were 157 differentially expressed lncRNAs in the 55-PHFBUD vs. 45-EPI comparison group, 802 differentially expressed lncRNAs in the 65-PHFDERM vs. 45-EPI comparison group, 670 differentially expressed lncRNAs in the 75-SHFINI vs. 45-EPI comparison group, 807 differentially expressed lncRNAs in the 65-PHFDERM vs. 55-PHFBUD comparison group, 628 differentially expressed lncRNAs in the 75-SHFINI vs. 55-PHFBUD comparison group, and 177 differentially expressed lncRNAs in the 75-SHFINI vs. 65-PHFDERM comparison group (Figure 1B). Our previous research found that at 45 days of the embryonic period of cashmere goats, the fetal skin forms a complete epidermal structure, and the morphology of hair follicles does not occur; at about 55 embryonic days, the primary hair follicles begin to appear, and the keratinocytes in the basal layer of the epidermis are arranged together in a palisading pattern to form the hair bud structure of the primary hair follicles; at about 65 embryonic days, the hair bud structure of the primary hair follicle grows downward into the dermis of the skin; at around 75 embryonic days, secondary hair follicle morphology begins to occur. The secondary hair follicle gradually matures and forms a complete hair follicle structure after 75 days [2,5].

Subsequently, we further screened and analyzed the lncRNAs that were differentially expressed during different embryonic periods. First, we set the three comparison groups of 55-PHFBUD vs. 45-EPI, 65-PHFDERM vs. 45-EPI, and 65-PHFDERM vs. 55-PHFBUD as Stage A, which had a total of 1051 lncRNAs related to primary hair follicle morphogenesis and development, and the three comparison groups of 75-SHFINI vs. 45-EPI, 75-SHFINI vs. 55-PHFBUD, and 75-SHFINI vs. 65-PHFDERM as Stage B, which had a total of 903 lncRNAs related to primary or secondary hair follicle morphogenesis and development. The common part of Stage B and Stage A was removed from Stage B, and the remaining 158 lncRNAs were regarded as important lncRNAs associated with secondary hair follicle morphogenesis and development (Figure 1A). Among the 158 lncRNAs related to the morphogenesis of the secondary hair follicle, we found that a lower expression (*p* < 0.05) of the lncRNA MSTRG.20890.1 occurred on d 75-SHFINI of the embryonic stage. Subsequently, we further verified its expression in the skin at different embryonic stages. Consistent with the sequencing results, the expression of lncRNA MSTRG.20890.1 was lower in the embryonic stage of 75-SHFINI (Figure 1F). Therefore, we chose this lncRNA for subsequent research.

### 3.2. LncRNA MSTRG.20890.1 Inhibits the Proliferation and Migration of Dermal Fibroblasts

LncRNA MSTRG.20890.1 is an RNA transcribed from the intron region of the *ZNF385D* gene with a length of 11,217 bp, and it is located at chrNC_030834.1: 1274805-1286021 (Figure 1A). We used the CPC 1.0 and CNCI 2.0 to analyze the coding ability of lncRNA MSTRG.20890.1. The results showed that the scores of CPC and CNCI were 0.274 and −0.043 respectively, indicating that lncRNA MSTRG.20890.1 cannot encode proteins. Subsequently, we constructed three pairs of lncRNA MSTRG.20890.1 interference vectors, respectively (Supplementary File S1), and transfected lncRNA MSTRG.20890.1-sh1/2/3 into dermal fibroblasts by lentivirus. As previously reported, hair follicle morphogenesis and development are a result of the continuous proliferation and differentiation of dermal fibroblasts and epithelial cells. Furthermore, dermal fibroblasts eventually form the dermal papilla structure through constant proliferation and differentiation, which is the signaling hub for the cyclic growth and regeneration of the hair follicle, so dermal fibroblasts were chosen for the subsequent experiments [4,5]. qRT-PCR was used to detect the interference efficiency of the vector. Vectors sh1, sh2 and sh3 could all significantly inhibit the expression of lncRNA MSTRG.20890.1, among which sh2 had the best interference efficiency (Figure 1G). Therefore, the lncRNA MSTRG.20890.1-sh2 interference cell line was selected for the subsequent experiments.

Firstly, the effect of lncRNA MSTRG.20890.1 interference on dermal fibroblasts was detected by flow cytometry. The results showed that after interfering with lncRNA MSTRG.20890.1, the apoptotic dermal fibroblasts were smaller than the control group (*p* ≤ 0.01) (Figure 1C). In addition, we used CCK8 and EDU to detect the proliferation of lncRNA MSTRG.20890.1-sh cell lines respectively. The results showed that after the interference of lncRNA MSTRG.20890.1, the number of EDU-positive cells of dermal fibroblasts increased significantly, and the proliferation ability of cells was higher (Figure 1D,E). The above experimental results indicate that after interfering with lncRNA MSTRG.20890.1, it could promote the proliferation ability of dermal fibroblasts and inhibit the apoptosis of cells. Subsequently, we further investigated the promoting effect of lncRNA MSTRG.20890.1-sh on cell proliferation. It was found that after the interference of lncRNA MSTRG.20890.1, the number of cells in the S phase increased (*p* ≤ 0.05), while the proportions of cells in the G1 phase and G2/M phase were significantly reduced, indicating that lncRNA MSTRG.20890.1-sh achieved cell proliferation by increasing the proportion of cells in the S phase and blocking the G1 phase and G2/M phase (Figure 1I). Throughout the entire process of hair follicle development, as the structure of hair follicles continues to mature, dermal fibroblasts not only proliferate and differentiate but also continuously penetrate the dermis to form the dermal papilla structure of hair follicles. Therefore, the scrape motility assay was used to explore the effect of lncRNA MSTRG.20890.1 on the migration ability of dermal fibroblasts. We found that the migration ability of dermal fibroblasts was strengthened after transfection of lncRNA MSTRG.20890.1-sh (*p* ≤ 0.05) (Figure 1H). The comprehensive results of the above experiments indicate that lncRNA MSTRG.20890.1-sh could improve the proliferation and migration ability of dermal fibroblasts. At the same time, its promoting effect on the proliferation of dermal fibroblasts might be achieved by increasing the proportion of cells in the S phase.

### 3.3. LncRNA MSTRG.20890.1 Can Participate in the Morphogenesis and Development of Secondary Hair Follicles by Binding to Chi-miR-24-3p

**Figure 2:**
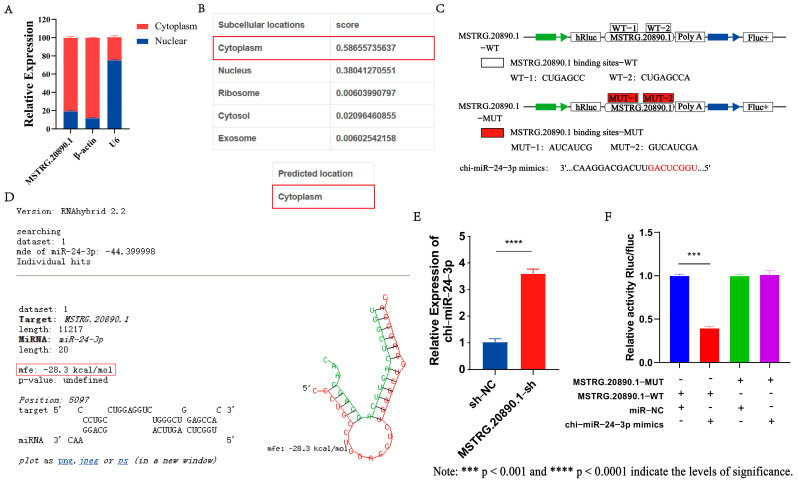
LncRNA MSTRG.20890.1 acts as a sponge for chi-miR-24-3p in cashmere goat. (**A**) Detection of lncRNA MSTRG.20890.1 expression in the nucleus and cytoplasm of dermal fibroblasts. (**B**) lncLocator software predicts the distribution of lncRNA MSTRG.20890.1 in cells. (**C**) Schematic diagram of wild/mutant lncRNA MSTRG.20890.1 luciferase reporter vector construction. (**D**) RNAhybrid (v2.1.2) software predicts the sequence of lncRNA MSTRG.20890.1 binding site to chi-miR-24-3p. (**E**) Relative expression of chi-miR-24-3p after transfection of dermal fibroblasts with lncRNA MSTRG.20890.1-sh. (**F**) Dual-luciferase reporter gene system to detect target binding of lncRNA MSTRG.20890.1 to chi-miR-24-3p.

The location of lncRNA in the cell determines its function. Studies have shown that lncRNAs in the cytoplasm can regulate gene expression by binding to miRNAs [25]. Therefore, lncLocator 1.0 software and isolation of cytoplasmic and nuclear RNA experiments were used to predict and analyze the location of lncRNA MSTRG. 20890.1 in cells. The results showed that it was mainly expressed in the cytoplasm (Figure 2A,B). Subsequently, we performed bioinformatics analysis using the TargetScan and miRanda database and found that the lncRNA MSTRG.20890.1 has a potential binding site with chi-miR-24-3p. Further, the RNAhybrid (v2.1.2) software was used to obtain the binding site sequence information between lncRNA MSTRG.20890.1 and chi-miR-24-3p. Figure 2D shows that the free energy of lncRNA MSTRG.20890.1 binding to chi-miR-24-3p was less than −20 kcal/mol, indicating a strong binding ability. The expression of chi-miR-24-3p in the lncRNA MSTRG.20890.1-sh cell line was detected, and it was found that this miRNA was lowly expressed in the cell line (*p* ≤ 0.001) (Figure 2E). Further, we constructed wild-type and mutant lncRNA MSTRG.20890.1 dual-luciferase reporter plasmids (Figure 2C), and a dual-luciferase reporter gene assay was performed to validate the target binding relationship between lncRNA MSTRG.20890.1 and chi-miR-24-3p. The results showed that the chi-miR-24-3p mimic reduced the fluorescence activity of the lncRNA MSTRG.20890.1-WT luciferase reporter gene (*p* ≤ 0.001), while the mutant type showed no significant change, indicating that there was indeed a targeted binding relationship between lncRNA MSTRG.20890.1 and chi-miR-24-3p (Figure 2F).

### 3.4. Chi-miR-24-3p Can Counteract the Promoting Effect of LncRNA MSTRG.20890.1-sh on the Proliferation and Migration of Dermal Fibroblasts

**Figure 3:**
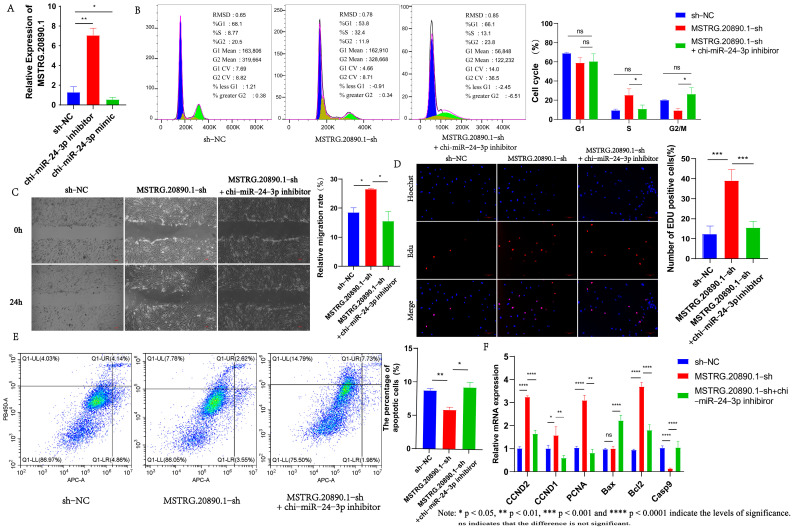
Chi-miR-24-3p can reverse the effect of lncRNA MSTRG.20890.1 on the cell phenotype of dermal fibroblasts. (**A**) The expression of lncRNA MSTRG.20890.1 was detected in chi-miR-24-3p interference/overexpression cell lines. (**B**) The cell cycle of lncRNA MSTRG.20890.1-sh cell line was detected after adding chi-miR-24-3p inhibitor. (**C**) The migration of lncRNA MSTRG.20890.1-sh cell line was detected after adding chi-miR-24-3p inhibitor. (**D**) lncRNA MSTRG.20890.1-sh cell line was added with chi-miR-24-3p inhibitor to detect cell proliferation. (**E**) The apoptosis of lncRNA MSTRG.20890.1-sh cell line was detected after adding chi-miR-24-3p inhibitor. (**F**) Expression of marker genes for cell proliferation/apoptosis.

To further explore the interaction between lncRNA MSTRG.20890.1 and chi-miR-24-3p in dermal fibroblasts, rescue experiments were carried out by co-transfection of lncRNA MSTRG.20890.1-sh and chi-miR-24-3p inhibitor. First, the expression of lncRNA MSTRG.20890.1 was detected in chi-miR-24-3p inhibitor and chi-miR-24-3p mimic cell lines, respectively. The expression of lncRNA MSTRG.20890.1 was increased after chi-miR-24-3p interference (*p* ≤ 0.01). On the contrary, its expression was reduced in chi-miR-24-3p overexpression cell lines (*p* ≤ 0.05) (Figure 3A). LncRNA MSTRG.20890.1-sh can promote the proportion of EDU-positive cells in dermal fibroblasts (*p* ≤ 0.001) and increase the number of cells in the S phase (*p* ≤ 0.05). However, when lncRNA MSTRG.20890.1-sh was co-transfected with chi-miR-24-3p inhibitor, the number of EDU-positive cells and S phase cells was significantly reduced (Figure 3B,D). Meanwhile, we found that lncRNA MSTRG.20890.1-sh could significantly promote the expression of cell proliferation-related genes such as *PCNA*, *CCND1*, and *CCND2*. However, after adding chi-miR-24-3p inhibitor in this cell line, the expression of proliferation-related genes was significantly decreased (relative to the experimental group lncRNA MSTRG.20890.1-sh) (Figure 3F). At the level of apoptosis, we found that apoptosis in dermal fibroblasts was promoted when chi-miR-24-3p inhibitor was added to the lncRNA MSTRG.20890.1-sh cell line and that this promotion counteracted the inhibition of apoptosis by the lncRNA MSTRG.20890.1-sh (*p* ≤ 0.05) (Figure 3E). At the same time, we found that when chi-miR-24-3p inhibitor was added to lncRNA MSTRG.20890.1-sh cell line, the mRNA expression levels of pro-apoptosis genes such as *Bax* and *Casp9* were significantly increased, while the mRNA expression levels of anti-apoptosis gene *Bcl2* were significantly decreased (Figure 3F). In terms of cell migration, lncRNA MSTRG.20890.1-sh promoted the migration of dermal fibroblasts, and this promoting effect was reversed after co-transfection with chi-miR-24-3p inhibitor (*p* ≤ 0.05) (Figure 3C). In conclusion, the above results indicate that the promoting effect of lncRNA MSTRG.20890.1-sh on the proliferation and migration of dermal fibroblasts can be rescued by chi-miR-24-3p inhibitor.

### 3.5. ADAMTS3 Can Target and Bind to Chi-miR-24-3p to Participate in the Morphogenesis and Development of Secondary Hair Follicles

**Figure 4:**
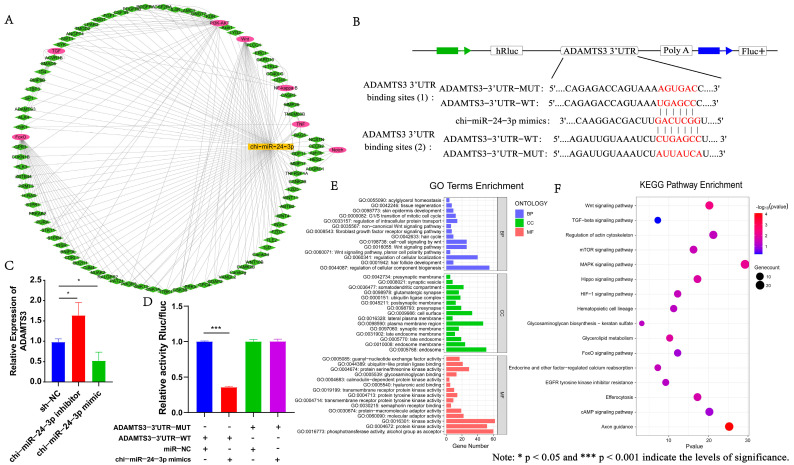
Prediction and analysis of chi-miR-24-3p target gene. (**A**) Construction of chi-miR-24-3p-mRNA regulatory network. (**B**) Schematic diagram of wild-type/mutant-type ADAMTS3-3′UTR luciferase reporter vector construction. (**C**) Detection of *ADAMTS3* expression in chi-miR-24-3p interference/overexpression dermal fibroblast cell lines. (**D**) Targeted binding of chi-miR-24-3p toADAMTS3-3′UTR was detected. (**E**) GO enrichment analysis of chi-miR-24-3p target genes. (**F**) KEGG enrichment analysis of chi-miR-24-3p target genes.

To further determine the potential regulatory mechanism, the TargetScan and miRanda databases were used to predict the target genes of chi-miR-24-3p. We found that chi-miR-24-3p has a targeting relationship with 1476 genes, and its regulatory network is shown in Figure 4A. Subsequently, we performed enrichment analysis for target genes of chi-miR-24-3p. The results showed that these target genes were enriched into signaling pathways related to hair follicle genesis and development, such as Wnt, TGF-β, and Fox0 (Figure 4E,F). Therefore, we selected the *ADAMTS3* gene enriched in the TGF-β signaling pathway for subsequent experimental verification and analysis.

First, we verified the targeting relationship between chi-miR-24-3p and *ADAMTS3* using the dual-luciferase reporter gene system. The wild-type and mutant plasmids of the 3′UTR region of *ADAMTS3* were constructed respectively (Figure 4B), and the wild-type and mutant plasmids were transfected into 293T cells with chi-miR-24-3p mimic, respectively. The results of the dual-luciferase reporter system showed that the chi-miR-24-3p mimic reduced the fluorescence activity of the ADAMTS3-3′UTR-WT luciferase reporter gene (*p* ≤ 0.001), while the mutant type did not change, indicating that chi-miR-24-3p indeed has a targeting binding site with *ADAMTS3* (Figure 4D). Subsequently, *ADAMTS3* expression was detected in the chi-miR-24-3p interference/overexpression dermal fibroblast cell lines. The results showed that interference with chi-miR-24-3p could increase the expression level of *ADAMTS3* (*p* ≤ 0.05), while overexpression of chi-miR-24-3p could inhibit the expression of *ADAMTS3* (*p* ≤ 0.05) (Figure 4C). The above results indicate that there is indeed a conserved target of chi-miR-24-3p in the *ADAMTS3,* and this targeted binding site can inhibit the expression of the *ADAMTS3* gene.

### 3.6. ADAMTS3 Inhibits the Proliferation and Migration of Dermal Fibroblasts

**Figure 5:**
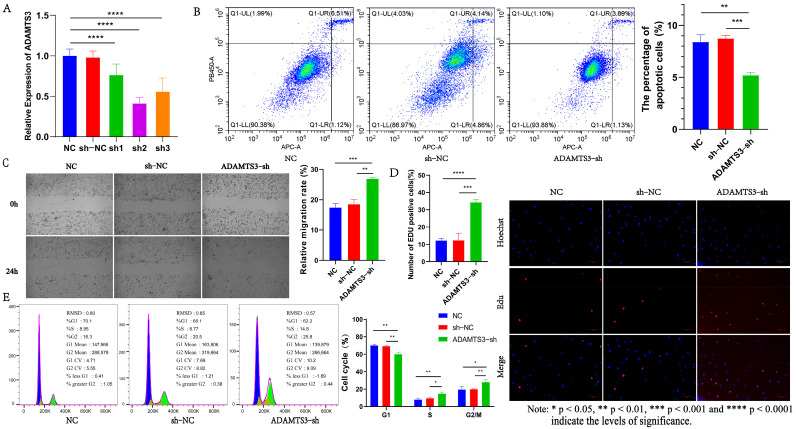
Functional analysis of *ADAMTS3* in dermal fibroblasts. (**A**) Screening of *ADAMTS3* interference vector. (**B**) The apoptosis of dermal fibroblasts was detected after *ADAMTS3* interference. (**C**) Migration ability of ADAMTS3-sh cell line. (**D**) EDU was used to detect the proliferation of ADAMTS3-sh cell line. (**E**) Cell cycle determination of ADAMTS3-sh cell line.

**Figure 6:**
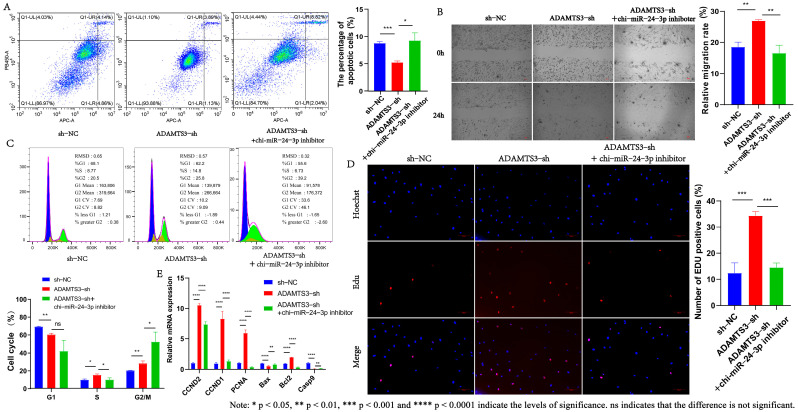
Chi-miR-24-3p can reverse the effect of *ADAMTS3* on the cell phenotype of dermal fibroblasts. (**A**) The apoptosis of ADAMTS3-sh cell line was detected after adding chi-miR-24-3p inhibitor. (**B**) The migration of ADAMTS3-sh cell line was detected after adding chi-miR-24-3p inhibitor. (**C**) The cell cycle of ADAMTS3-sh cell line was detected after adding chi-miR-24-3p inhibitor. (**D**) The proliferation of ADAMTS3-sh cell line was detected after adding chi-miR-24-3p inhibitor. (**E**) Expression of marker genes for cell proliferation/apoptosis.

The ADAMTS proteinase family was first identified in mice, and this family is similar in structure to ADAM enzymes [26,27]. This family can be divided into four subclasses, among which *ADAMTS3* belongs to the second subclass and is an important gene in the TGF-β signaling pathway [27]. Therefore, we constructed three *ADAMTS3* interference vectors and transfected them into dermal fibroblasts by lentiviral transfection (Supplementary File S1). qRT-PCR was used to detect the interference efficiency of ADAMTS3-sh1/sh2/sh3. The results showed that the interference efficiency of ADAMTS3-sh2 was the best, so sh2 was selected for the subsequent experiments (*p* ≤ 0.001) (Figure 5A). Firstly, the results of proliferation and apoptosis experiments showed that ADAMTS3-sh could inhibit apoptosis of dermal fibroblasts (*p* ≤ 0.01) and promote cell proliferation (*p* ≤ 0.001) (Figure 5B,D). The expression of cell proliferation and apoptosis marker genes in the cell line was detected, and it was found that ADAMTS3-sh could promote the expression of cell proliferation-related marker genes and inhibit the expression of apoptosis-related genes (Figure 6E). In addition, we further explored the effect of *ADAMTS3* on the cell cycle by DNA staining. We found that the knockdown of *ADAMTS3* increased the proportion of cells in the S phases (*p* ≤ 0.05) (Figure 5E). The results of the cell migration experiment showed that when ADAMTS3-sh was transferred into dermal fibroblasts, its cell migration ability increased significantly (*p* ≤ 0.01) (Figure 5C). The results showed that knockdown of *ADAMTS3* enhanced the proliferation and migration of dermal fibroblasts, and decreased the apoptosis of dermal fibroblasts. It should be noted that the effects of ADAMTS3-sh and lncRNA MSTRG.20890.1-sh on the phenotype of dermal fibroblasts are consistent, and this phenomenon exactly conforms to the ceRNA hypothesis.

### 3.7. LncRNA MSTRG.20890.1 Inhibits the Proliferation and Apoptosis of Dermal Fibroblasts Through the Chi-miR-24-3p/ADAMTS3 Signaling Axis

Based on previous studies, we found that chi-miR-24-3p can bind to lncRNA MSTRG.20890.1, and chi-miR-24-3p inhibitor can counteract the promoting effect of lncRNA MSTRG.20890.1-sh on the proliferation and migration of dermal fibroblasts, as well as the inhibiting effect on apoptosis. Therefore, we hypothesized that the chi-miR-24-3p inhibitor could also act as an inhibitor of ADAMTS3-sh to counteract the effect of ADAMTS3-sh on the phenotype of dermal fibroblasts. To prove this hypothesis, we performed co-transfection of ADAMTS3-sh and chi-miR-24-3p inhibitor and verified their relationship at the cellular level by rescue assay. The results of the apoptosis assay showed that the ADAMTS3-sh cell line with chi-miR-24-3p inhibitor can counteract inhibition of apoptosis induced by ADAMTS3-sh (*p* ≤ 0.05) (Figure 6A). The changes in cell proliferation and migration were detected using the EDU method and the scrape motility assay. It can be seen from Figure 6B,D that after chi-miR-24-3p inhibitor was added to ADAMTS3-sh cell line, EDU-positive cells were reduced (*p* ≤ 0.001), and cell migration ability was also decreased (*p* ≤ 0.01). Subsequently, we further explored the effect of chi-miR-24-3p inhibitor on the ADAMTS3-sh dermal fibroblast cell line through the DNA staining method. We found that the increase in the proportion of cells in the S phase caused by ADAMTS3-sh was alleviated by the addition of chi-miR-24-3p inhibitor to the ADAMTS3-sh cell line (*p* ≤ 0.05) (Figure 6C). Meanwhile, we found that ADAMTS3-sh can promote the expression of cell proliferation-related marker genes and inhibit the expression of apoptosis-related genes. When ADAMTS3-sh2 and chi-miR-24-3p inhibitor were co-transfected, the expression of cell proliferation-related marker genes was significantly inhibited, while the expression of apoptosis-related marker genes was significantly promoted (Figure 6E). The above results indicate that there is indeed a targeting relationship between chi-miR-24-3p and *ADAMTS3* in cells, and they jointly regulate the proliferation and migration of dermal fibroblasts.

## 4. Discussion

The cashmere goat is an outstanding livestock breed that has been shaped by long-term natural selection and artificial breeding, and it is a distinctive biological resource worldwide. The development of the cashmere goat breeding industry plays an irreplaceable role in ensuring the lives of residents and promoting the development of the agricultural economy. With the rapid development of molecular biology technology, research on the hair follicles of cashmere goats has been gradually carried out. However, studies mainly focus on the periodic growth of hair follicles in cashmere goats, and research on the development of secondary hair follicles in the embryonic stage is still very scarce. In a previous study, we used high-throughput sequencing technology to construct the skin transcriptome database of cashmere goats at different embryonic stages and identified 1209 differentially expressed lncRNAs. In this study, according to the characteristics of the development of primary/secondary hair follicles in the embryonic period of cashmere goats, we finally found an RNA (lncRNA MSTRG.20890.1) with a length of 11,217 bp and no coding ability. It was significantly down-expressed on the 75-SHFINI day of the embryonic stage, and we guessed that it might play an important role in the morphogenesis of secondary hair follicles of cashmere goats. To prove this hypothesis, we further explored its influence on the phenotype of hair follicle cells and its regulatory mechanism.

Initially, lncRNAs were widely believed to have no biological function due to their lack of coding ability [28]. However, with the rapid development of biotechnology, more and more lncRNAs have been identified. For example, Nie [29] identified 62 significantly differentially expressed lncRNAs in sheep fetal skin samples by high-throughput sequencing technology, of which 26 were down-regulated. Among them, the lncRNAs interacted with BMP signaling (*SOSTDC 1*) and Wnt signaling (*Wnt16*, *SFRP1*) to affect epidermis and hair follicle placentation development. Zhou [30] constructed the transcriptome database of hair follicles in the anagen/catagen stage, and identified 1122 known lncRNAs and 403 novel lncRNAs. Among them, 173 lncRNAs were differentially expressed between the anagen stage and the catagen stage. Compared with other non-coding RNAs, lncRNA has more diverse regulatory mechanisms, and its regulatory role is ubiquitous in the whole genome, involving cell cycle, cell differentiation, and epigenetic regulation. Meanwhile, the regulatory mechanism of lncRNA is related to its location in the cell. The lncRNAs located in the cytoplasm can usually function through the ceRNA regulatory mechanism. Some studies have found that during the cyclic growth and development of hair follicles, lncRNAs often play an indirect regulatory role. For example, in the differentiation process of hair follicle stem cells, it was found that lnc5322 can regulate the expression of hair follicle-related transcription factors by targeting miR-21, thereby promoting the proliferation and differentiation of hair follicle stem cells [31]. In addition, PlncRNA-1 could mediate the Wnt/β-catenin signaling pathway through *TGF-β1* to regulate the proliferation and differentiation of hair follicle stem cell (HFSC) [32]. During the formation of dermal papilla structure, lncRNA-599547 can combine with chi-miR-15b-5p to regulate the expression of the *Wnt10* gene, thereby affecting hair follicle growth induced by dermal papilla [33]; lncRNA-XIST can activate Hedgehog signaling by regulating miR-424, thereby affecting dermal papillae to induce hair follicle reconstruction [13].

The hair follicle is an organ with regenerative functions. Its development begins during the embryonic period and is completed after birth [4]. The formation of mammalian hair follicles relies on a series of signal transduction between the epidermis and dermis, which induces the orderly proliferation and differentiation of epithelial cells and dermal fibroblasts, and then forms a complete hair follicle structure [34,35]. The dermal papilla is the “receive/send signal” center, which can ensure the normal growth and regeneration of hair follicles. In the process of dermal papilla formation, dermal fibroblasts eventually form mature dermal papilla structures through directional migration and continuous proliferation and differentiation [4]. Therefore, in this study, we focused on the effects of genes on the migration and proliferation of dermal fibroblasts. First, we constructed the lncRNA MSTRG.20890.1 interfering dermal fibroblasts cell line and used flow cytometry, scrape motility assay, and other experiments to further detect its effect on dermal fibroblasts. We found that after interfering with lncRNA MSTRG.20890.1, the proliferation and migration abilities of dermal fibroblasts significantly increased, and the apoptotic ability decreased. At the same time, through the detection of DNA staining and proliferation-related gene expression, we further demonstrated that this promoting effect on cell proliferation was achieved by the expression of proliferation-promoting marker genes and increasing the proportion of S-phase cells. Subsequently, we further explored the regulatory mechanism of lncRNA MSTRG.20890.1. Since the distribution of lncRNA in cells is related to its regulatory mechanism, we further analyzed its distribution in dermal fibroblasts. The prediction of lncLocator software and the results of nuclear-cytoplasmic separation experiments show that lncRNA MSTRG.20890.1 is mainly distributed in the cytoplasm. Therefore, we used the TargetScan and miRanda databases to initially construct the lncRNA MSTRG.20890.1-miRNA-mRNA regulatory network, and finally selected the lncRNA MSTRG.20890.1-chi-miR-24-3p/ADAMTS3 signaling axis enriched in the TGF-β signaling pathway for subsequent exploration.

The TGF-β signaling pathway mainly participates in processes such as the normal development and immune response of organisms by regulating processes such as cell differentiation, and migration [36]. TGF-β signaling is regarded as a key molecule for hair follicle development, differentiation, and downward growth. The deficiency of TGF-β signaling will cause incomplete epidermal development and the inhibition of the downward growth process of hair follicles [37,38]. Previous studies have also shown that *ADAMTS3* is involved in the development of hair follicles [16]. In this study, we found that the knockdown of *ADAMTS3* in dermal fibroblasts significantly increased the proliferation and migration ability of the cells, while significantly decreasing the apoptosis ability of the cells. This result is similar to that after lncRNA MSTRG.20890.1 interference, which is consistent with the ceRNA regulatory mechanism. To further validate the lncRNA MSTRG.20890.1/chi-miR-24-3p/ADAMTS3 regulatory network, we performed experiments using the dual luciferase reporter gene system and rescue assay. The results showed that chi-miR-24-3p mimics could reduce the fluorescence activities of lncRNA MSTRG.20890.1-WT and ADAMTS3-3′UTR-WT luciferase reporter genes, but the mutant type had no change. In the rescue experiment, the lncRNA MSTRG.20890.1-sh cell line and ADAMTS3-sh cell line were added with chi-miR-24-3p inhibitor, respectively. We found that the promoting effects of lncRNA MSTRG.20890.1-sh and ADAMTS3-sh on the proliferation and migration of dermal fibroblasts were counteracted by the chi-miR-24-3p inhibitor. The above results confirmed our conjecture that lncRNA MSTRG.20890.1 indeed inhibited the proliferation and migration of dermal fibroblasts through the chi-miR-24-3p/ADAMTS3 signaling axis, thereby inhibiting dermal papillae formation and secondary hair follicle morphogenesis.

In conclusion, we discovered a lncRNA MSTRG.20890.1 transcribed from the intron region of the *ZNF385D* gene, which was significantly lowly expressed in the skin at 75-SHFINI days of the embryo. Furthermore, lncRNA MSTRG.20890.1 can inhibit the proliferation and migration of dermal fibroblasts by competitively binding chi-miR-24-3p with *ADAMTS3*, thereby inhibiting the formation of dermal papilla structures and the morphogenesis of secondary hair follicles. The results of this study will help to further analyze the molecular regulatory mechanisms during hair follicle morphogenesis and development, which is of great significance to enhancing the productive traits of fiber-producing livestock and expediting the cultivation of new cashmere goat varieties.

## 5. Conclusions

**Figure 7:**
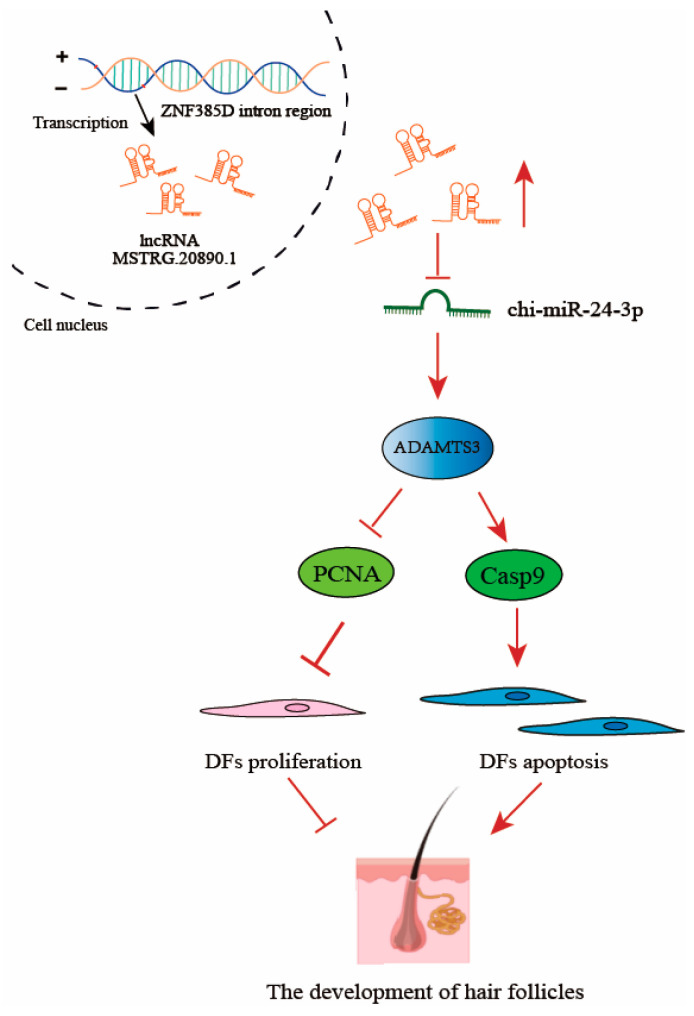
Diagram of the lncRNA MSTRG.20890.1/chi-miR-24-3p/ADAMTS3 regulatory mechanism.

In this study, we discovered 158 lncRNAs that were differentially expressed during the morphogenesis of secondary hair follicles in cashmere goats. Among them, lncRNA MSTRG.20890.1 was significantly lowly expressed at 75-SHFINI day. The 75th day is the critical period for secondary hair follicle morphogenesis. Therefore, we further studied the effect of lncRNA MSTRG.20890.1 on dermal fibroblasts. The results showed that after interfering with lncRNA MSTRG.20890.1, the proliferation and migration abilities of dermal fibroblasts significantly increased, and the apoptotic ability was inhibited. Further, we demonstrated by rescue experiments that lncRNA MSTRG.20890.1 was inhibiting the proliferation and migration of dermal fibroblasts through the chi-miR-24-3p/ADAMTS3 signaling axis, which in turn inhibited the formation of dermal papilla structures and secondary follicle morphogenesis (Figure 7).

## Data Availability

The raw data of Inner Mongolia cashmere goat skin transcriptome sequencing were submitted to the SRA database under accession numbers (SRR13306938-6949).

## Supplementary Materials

The following supporting information can be downloaded at: https://www.mdpi.com/article/10.3390/genes15111392/s1, Supplementary File S1: Sequence information of interference and overexpression vectors. Supplementary File S2: The primers of qRT-PCR for genes.
