# Supplementary material for: Role of LncRNA MSTRG.20890.1 in Hair Follicle Development of Cashmere Goats
Source: Genes (Basel). 2024 Oct 29;15(11):1392. doi: 10.3390/genes15111392 (PMC11593464; doi:10.3390/genes15111392)
Supplement: Supplementary file 1 [file genes-15-01392-s001.zip › genes-3238143-supplementary/Supplementary material/Supplementary 1.pdf]

LncRNA MSTRG.20890.1-sh sequence information

siRNA1：CCTGGAATTATGAGAGGCATCTTAA

siRNA2：AGACGGACCTTAGTCGGCAAAGTAA

siRNA3：GATCCAGCGGATGTTGGCAATTTGA

ADAMTS3-sh sequence information

siRNA1：CAAACACCACTGGTTGCCATATGAA

siRNA2：CAGTAAATCCAATGGTGCTAACTTA

siRNA3：TCTCCGGTGCCTGGGAATATA

chi-miR-24-3p mimic sequence information

GAAGTCAAGCAAGACTATGTCAGCGGTAGATGATGTGTGAGTAGAGCTGGGCGCTTTCAAAGGAGACGTCCTCTTGTGATTT

CCGAGCACGGTGAACTCTCTCTTGTATTTGCAGTCCAGGTCTGTGTCTTCTGCAGCGCCAGTGGAACGAGGCCGAGCGAGC

TCCCAGCCGAGGCGCTGCTTCTCCGGGCTGTCAGTTGGACCCGCCCTCCGGTGCCTACTGAGCTGATATCAGTTCTCATTTTA

CACACTGGCTCAGTTCAGCAGGAACAGGAGTCGAGCCCTAGAGCAAAGCCTTCCTGTTTGTAAGTGCCCAGAGGCCTGGG

AGCTGAGACTGCAGCTGCGTGAGGACCGGCCTGAGCGCCCGCAGCACAGCTGACCGGCAGCGGTGGTGGCCCCTCTGTAT

GTGTCCTGTGTGTGGGTGTCGGTACAGTCAGAGTTCTGTTGATACAAGAGAAATGAGCTTCCACCCGAAAAGGC

chi-miR-24-3p inhibitor sequence information

GTTCCTGCTTCCTGAGCCATATACGTTCCTGCTTCCTGAGCCAACATCGTTCCTGCTTCCTGAGCCATCTTCAGTTCCTGCTTC

CTGAGCCA
